# Revolutionizing Gastrointestinal Disorder Management: Cutting-Edge Advances and Future Prospects

**DOI:** 10.3390/jcm13133977

**Published:** 2024-07-08

**Authors:** Chahat Suri, Babita Pande, Tarun Sahu, Lakkakula Suhasini Sahithi, Henu Kumar Verma

**Affiliations:** 1 Department of Oncology, Cross Cancer Institute, University of Alberta, Edmonton, AB T6G 1Z2, Canada; csuri@ualberta.ca; 2 Lung Health and Immunity, Helmholtz Zentrum Munich, IngolstädterLandstraße 1, 85764 Oberschleißheim, 85764 Munich, Germany; 3 Department of Physiology, All India Institute of Medical Science, Raipur 492099, India; babitatime2014@gmail.com (B.P.); tarunsahu@aiimsraipur.edu.in (T.S.); 4 Department of Biotechnology, Guru Ghasidas Vishwavidyalaya, Bilaspur 495009, India; lakkakulasahithi@gmail.com

**Keywords:** gastrointestinal disorders, management, theraputics, diagnosis, artificial intelligence

## Abstract

In recent years, remarkable strides have been made in the management of gastrointestinal disorders, transforming the landscape of patient care and outcomes. This article explores the latest breakthroughs in the field, encompassing innovative diagnostic techniques, personalized treatment approaches, and novel therapeutic interventions. Additionally, this article emphasizes the use of precision medicine tailored to individual genetic and microbiome profiles, and the application of artificial intelligence in disease prediction and monitoring. This review highlights the dynamic progress in managing conditions such as inflammatory bowel disease, gastroesophageal reflux disease, irritable bowel syndrome, and gastrointestinal cancers. By delving into these advancements, we offer a glimpse into the promising future of gastroenterology, where multidisciplinary collaborations and cutting-edge technologies converge to provide more effective, patient-centric solutions for individuals grappling with gastrointestinal disorders.

## 1. Introduction

The group of gastrointestinal disorders [GI] is characterized by various combinations of motility disturbances, visceral hypersensitivity, changes in mucosal and immune function, changes in gut microbiota, and central nervous system processing [1]. These disorders include inflammatory bowel diseases [IBDs], functional gastrointestinal disorders [FGIDs] like irritable bowel syndrome [IBS], gastroesophageal reflux disease [GERD], peptic ulcers, colorectal cancer, and others [2]. IBDs encompass collection of chronic conditions that lead to inflammation in the digestive tract, significantly impacting quality of life through symptoms such as abdominal pain, diarrhea, rectal bleeding, and fatigue [3]. IBD affects millions of people worldwide, with a higher prevalence in Western countries [4,5]. The two conditions under IBD are, Crohn’s disease and ulcerative colitis. Despite sharing similarities, these conditions have distinct differences. Crohn’s disease can affect any part of the digestive tract, from the mouth to the anus [6,7] In Crohn’s disease, inflammation occur in patches, leading to ulcers and bowel wall thickening resulting in symptoms such as abdominal pain, diarrhea, weight loss, and malnutrition [8]. Complications may include fistulas or abscesses. On the other hand, ulcerative colitis primarily affects the colon and rectum [9]. The inflammation in ulcerative colitis is continuous and limited to the innermost lining of the colon, causing bloody diarrhea, abdominal pain, and a constant urge for bowel movements. Severe cases may lead to complications like toxic megacolon or colon cancer [10,11]. The exact causes of IBD remain unclear, but it is thought to involve a combination of genetic, environmental, and immune system factors. Genetics plays a significant role in individuals with family history of IBD. Several genes associated with increased susceptibility to IBD have been identified [12]. However, not all individuals with these genetic repertoires develop the disease, indicating the involvement of other factors. Consuming western diet, particularly processed foods, sedentary lifestyle, and exposure to certain infections or pollutants may increase IBD risk [13]. Smoking is a known risk factor for Crohn’s disease but appears to have a protective effect against ulcerative colitis. The immune system’s role is pivotal in IBD development [14]. In individuals with IBD, the immune system erroneously attacks the healthy cells in the digestive tract, causing inflammation, likely triggered by both genetic and environmental factors [15].

A comprehensive assessment of the patient’s medical history, a physical examination, and various tests are necessary to diagnose IBD. Blood tests can help to identify inflammation or anemia, while stool tests can detect infections or parasites. Endoscopic procedures, such as colonoscopy or sigmoidoscopy, allow direct examination of the digestive tract and the collection of tissue samples for further analysis. Imaging studies, including computed tomography [CT] scans or magnetic resonance imaging [MRI] scans, provide additional information about the extent and location of inflammation [16]. Differentiating between Crohn’s disease and ulcerative colitis is crucial. In some cases, making a definitive diagnosis may be challenging, necessitating additional tests or consultations with specialists [17].

Furthermore, the pathophysiological mechanisms underlying GI disorders remain incompletely understood. Nevertheless, the biopsychological model, which considers genetic, cultural, environmental, and psychological factors, suggests potential causes, including modifications of gut microbiota, gastrointestinal motility, and the gut-brain axis. These factors are also linked to low-grade inflammatory processes and visceral hypersensitivity. The pathophysiology of GI disorders is complex, but the biopsychological model provides a framework for understanding it [18]. The bidirectional communication pathways between the gut and the brain play a major role in the pathogenesis of GI disorders. The model also considers the interaction of psychosocial factors, genetics, environmental factors, diet, early life trauma, and disruptions in the gut-brain axis with functional GI disorders. Another mechanism for the pathogenesis of GI disorders is the direct infection of gastrointestinal cells with a virus [19]. The family of coronaviruses [CoVs], for instance, has a direct relationship with the health of the gut. SARS-CoV-2 [severe acute respiratory syndrome coronavirus 2] uses ACE2 [angiotensin-converting enzyme 2], as a receptor to enter cells, as ACE2 is expressed in the gastrointestinal tract [20,21]. This is why GI tract may be a target organ for the virus. Clinicians need to investigate the symptoms carefully to prevent and cure GI disorders. Patients with GI disorders manifest a wide range of symptoms depending on the specific condition they are experiencing. These symptoms can be mild, moderate, or severe, and may recur or persist over time. It is essential to note that many GI disorder share overlapping symptoms, making an accurate diagnosis reliant on a comprehensive evaluation by a healthcare provider. Individual experiencing persistent or severe gastrointestinal symptoms are advised to seek medical attention to understand the underlying cause and initiate appropriate treatment. Treatment guidelines include four major levels: educating the patient about the disorder, consultation, nutrition management, and drug treatment [22,23].

Medications play a crucial role in managing IBDs by reducing symptoms and maintaining remission. Depending on the type and severity of the condition, as well as the patient’s individual history, different medications can be prescribed [24]. Aminosalicylates [5-ASA drugs], are commonly used to treat mild to moderate forms of IBD, reduce inflammation in the lining of the digestive tract. Corticosteroids are potent anti-inflammatory medications that can provide rapid relief for individuals with moderate to severe IBD, by suppressing the immune system and reducing inflammation. However, due to their potential side effects, such as weight gain, mood swings, and bone thinning, corticosteroids are typically used for short-term treatment or as bridge therapy while waiting for other medications to take effect [25,26].

Immunomodulators, such as azathioprine or methotrexate, are utilised to suppress the immune system’s abnormal response in individuals with moderate to severe IBD. These medications can aid in inducing and maintaining remission, reducingthe need for corticosteroids, and preventing the recurrence of symptoms [27]. However, their effects may take several weeks or months to become noticeable. Biologic therapies represent a newer class of medications target specific molecules involved in the inflammatory process. Examples include infliximab, adalimumab, or vedolizumab, which are generally effective for individuals with moderate to severe IBD who have not responded well to other treatments. Biologics are administered either as intravenous infusions or subcutaneous injections and can induce and maintain remission, reduce the need for corticosteroids, and promote mucosal healing [28]. Antibiotics may be prescribed in specific cases of IBD, such as those involving infection or abscess formation. While antibiotics can help reduce inflammation and prevent complications, they are not typically used as long-term maintenance therapy [29].

Thus, managing GI disorders involves a multifaceted approach encompassing pre-endoscopic, endoscopic, and post-endoscopic interventions. Tailoring the management plan to the specific condition and patient’s needs is essential for optimizing outcomes and improving the quality of life of patients [30]. Patients suspected of GI bleeding, such as hematemesis, or melena, requires different treatments based on the etiology of the bleeding or disorder. Therefore, evaluation of the symptoms and vital signs is critical. The entire treatment history must be assessed, particularly in case of hypovolemic shock, rapid pulse rate, and high blood nitrogen level at the time of presentation [31].

This narrative literature review aims to discuss the various management strategies for GI disorders available worldwide. To identify better treatment practices, we have highlighted the knowledge gap, advancements in diagnostic and current treatment methods, discussed precision medicine, and addressed the current challenges and management recommendations. Furthermore, we also incorporated the side effects of different therapies and potential future prospects. The search includes studies related to gastrointestinal disorders and their management strategies, as well as advances in diagnosis and treatment approaches using search engines such as Google Scholar and PubMed.

## 2. Diagnostic Innovations: From Traditional to Precision Medicine

The progression of diagnostics for gastrointestinal disorders has been remarkable, transitioning from traditional methods to a technologically advanced precision medicine. This evolution has not only improved diagnostic accuracy and timeliness, but it has also enabled more personalized and patient-centered treatment. Historically, GI disorders were diagnosed primarily through clinical evaluation, which included the patient’s medical history and physical examination [32]. While these traditional methods remain important, they frequently lack the specificity required for precise diagnosis, particularly in complex conditions such as IBS. The diagnostic innovations along with their advantages, applications, and related GI disorders are summarized in Table 1.

X-rays, barium contrast studies, and computed tomography [CT] scans have all played important roles in detecting structural abnormalities in the gastrointestinal tract. However, these methods provide limited insight into functional and molecular aspects of diseases and involve exposure to ionizing radiation. The evolution of diagnostic technique has led to modern endoscopy, which has significantly improved diagnostic capabilities for GI disorders [33]. High-definition imaging, narrow-band imaging [NBI], and confocal laser endomicroscopy [CLE] have enhanced visualization and tissue characterization precision, allowing for early detection and targeted biopsies. Furthermore, the incorporation of non-invasive biomarkers, such as fecal occult blood testing [FOBT], serum markers [such as CA 19-9], and fecalcal protectin, has transformed the diagnostic process by providing more accessible and less invasive options for detecting conditions such as colorectal cancer and IBD [34]. Advances in molecular biology and genetics have introduced molecular diagnostics into gastroenterology. Technique such as polymerase chain reaction [PCR] and next-generation sequencing [NGS] allow for the detection of specific genetic mutations, aiding in the precise diagnosis for conditions such as hereditary colorectal cancer syndromes [35].

**Figure 1:**
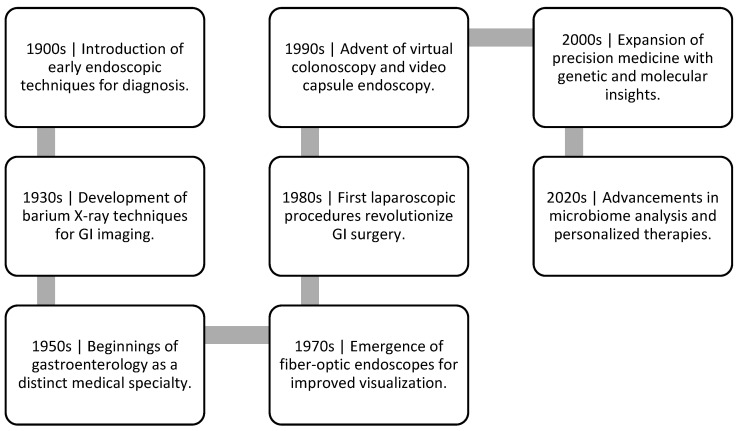
Timeline for the Gastrointestinal Care Advancement.

The COVID-19 pandemic has accelerated the adoption of telemedicine and remote monitoring tools in gastroenterology. Virtual consultations and wearable devices enable healthcare providers to remotely monitor patients, collect data, and make clinical decisions in real-time [16]. As illustrated in Figure 1, conventional endoscopy has been the gold standard for diagnosing and treating GI diseases. This technique involves inserting a flexible tube with a camera into the GI tract to visualize the lining of digestive system [36]. However, with the development of a biopsychological model of the disease, treatment has shifted its focus to the virtual modes of treatment targeting the brain-gut axis. Despite advances in understanding the brain-gut axis and its role in GI disorders, clinicians often persist in using the Cartesian split for treatment [37]. Many gastroenterologists continue to ignore or deny the role of the central nervous system [CNS] in disorders such as functional or IBD or chronic oesophageal disorders including functional heartburn [FH] and functional chest pain [FCP] and dismiss psychological comorbidity as neurotic or hysterical behaviour unrelated to the disease process. Conversely, psychiatrists have been more accepting of the concept of somatization, viewing the manifestation of CNS pathology as more physical symptoms, but have yet to establish a basis for various symptom-based somatic syndromes, especially concerning the GI tract [1].

Several advancements in the treatment of GI disorders has been incorporated into clinical practice [37]. However, many patients present risk factors such as advanced age and multiple significant comorbidities associated with poorer outcomes. Although the cause of GI disorders remains unknown, guidelines often classify them as variants and subtypes until endoscopy is performed. Personalized treatment is deemed the optimal approach to managing patients. Nonetheless, symptom overlap is a common complexity in these cases. As a result, two or more FGIDs, as well as other medically unexplained conditions such as chronic fatigue syndrome or fibromyalgia, may coexist. The frequency and severity of gastrointestinal symptoms, along with the number of co-existing FGIDs, all contribute to the prevalence of anxiety and depression [1,38].

The possible targeted interventions include therapies that reduce immune activation, block the release of histamine or utilise specific microbial treatments, along with dietary changes to eliminate relevant food antigens. Only by identifying causation, we can hope to anticipate a cure. Clinical findings indicate that the brain’s close interaction with the GI tract leads to a significant alteration in the bidirectional relationships between brain function and emotional states, likely to cause a wide range of functional and organic GI disorders. Influences from the brain on the gut may be more important in functional GI disorders, whereas influences from gut inflammation on brain function are more important in organic GI disorders [1].

Endoscopic techniques have broadened the scope of minimally invasive treatments. Endoscopic mucosal resection [EMR] and endoscopic submucosal dissection [ESD] have allowed for the removal of early gastrointestinal cancers and large polyps without surgery. Endoscopic retrograde cholangiopancreatography [ERCP] and endoscopic ultrasound-guided fine-needle aspiration [EUS-FNA] have established better diagnosis and management of pancreatic and biliary diseases with minimal invasiveness [39]. Metabolomic research has identified biomarkers in bodily fluids that correlate with disease status. For example, measuring elevated fecal calprotectin levels has become a non-invasive method for assessing intestinal inflammation in IBD patients [40]. Technological advances in metagenomics have revealed the importance of gut microbiota in gastrointestinal health. As seen in case of fecal microbiota transplantation for recurrent *Clostridium* complicated infection, microbiome analysis helps to identify dysbiosis and guide therapeutic interventions [41].

**Figure 2:**
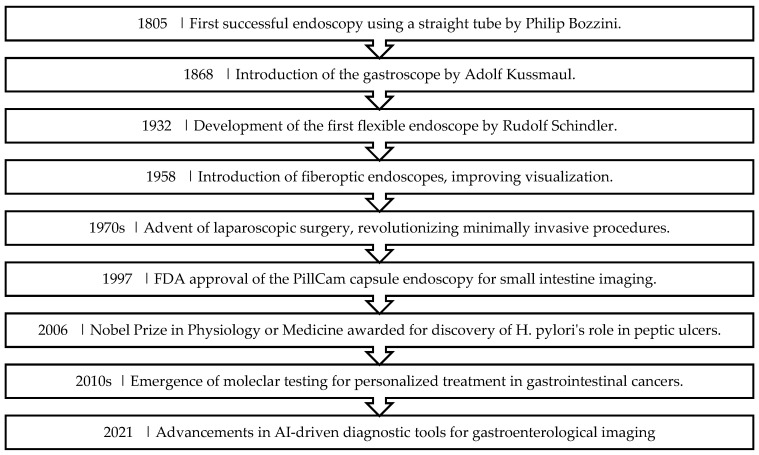
The Precision Medicine Paradigm.

Endoscopy has long been used to diagnose and treat digestive problems. Significant advancements inin endoscopic technology from 1805 to 2021, transitioning from straight tube to AI driven diagnostic methods [as depicted in Figure 2], have enhanced the diagnostic precision and facilitated minimally invasive approaches for therapeutic interventions. These advancements have improved the early detection of gastrointestinal conditions and treatment of GI cancers [42,43].

New endoscopic techniques such as chromoendoscopy, narrow-band imaging [NBI], flexible spectral imaging color enhancement [FICE], magnification endoscopy, confocal laser endomicroscopy [CLE], and i-SCAN have upgraded the sensitivity and specificity of cancer and precancerous lesion detection. These modern endoscopic technologies have improved the prognosis for gastrointestinal cancers by enabling early detection and treatment [43]. These developments marked a shift toward precision medicine, which aims to tailor medical treatment according to each patient’s unique characteristics. High definition [HD] imaging systems in modern endoscopes provide exceptional clarity and detail, allowing physicians to visualize subtle mucosal abnormalities. High-definition endoscopy has been instrumental in detecting colorectal polyps and early-stage gastrointestinal cancers [44]. NBI is an optical enhancement technique that uses specific light wavelengths to improve the visibility of mucosal structures and vasculature. This technology aids in distinguishing benign from malignant lesions, thereby improving diagnostic accuracy during endoscopy [45].

Endoscopic ultrasound [EUS] combines endoscopy with ultrasound technology to provide high-resolution imaging of the gastrointestinal wall and adjacent structures. It is particularly valuable in staging gastrointestinal cancers and assessing the depth of invasion, lymph node involvement, and vascular invasion [46].

The use of biomarkers in the early diagnosis, monitoring, and treatment of GI disorders marks a significant advance in personalized medicine. Biomarkers, which are indicators of biological processes or disease states, are critical in improving the precision and efficacy of patient care across a wide range of gastrointestinal conditions [47]. Biomarkers have transformed disease monitoring in chronic GI conditions such as IBD. Alanine aminotransferase [ALT] and aspartate aminotransferase [AST] are enzymes that are used to assess liver function, including non-alcoholic fatty liver disease [NAFLD]. Therefore, biomarkers are crucial in customising treatment approaches for gastrointestinal disorders [48].

Biomarkers like fecalcal protectin and C-reactive protein [CRP]act as indicators of inflammation in disorders such as IBD, aiding in medication optimization and disease monitoring [47]. Serum markers such as carcinoembryonic antigen [CEA] and carbohydrate antigen 19-9 [CA 19-9] assistin the diagnosis and monitoring of gastrointestinal cancers, enabling clinicians to make informed treatment and follow-up decisions [49]. Overall, endoscopic techniques, imaging modalities, and biomarker identification have improved the diagnosis and management of gastrointestinal disorder, allowing for earlier detection and more effective treatment.

High-throughput DNA sequencing techniques, such as next-generation sequencing [NGS], are effective in identifying genetic variants associated with GI disorders. NGS can identify gene mutations such as APC, SMAD4, and MSH2in patients with familial adenomatous polyposis [FAP], assisting in early detection and personalized risk assessment. Transcriptomic and proteomic profiling have enabled the identification of disease-specific signatures. In IBD, gene expression profiling can distinguish between active and dormant disease states, guiding treatment decisions [50].

**Table 1:** Diagnostic Innovations in Gastrointestinal Disorder Management.

Diagnostic Method	Advantages	Applications	Associated Disease Types	Reference
Advanced Endoscopy	Early detection of lesions and abnormalities	Gastrointestinal cancer screening, Precise localization of lesions	Gastrointestinal cancers	[36]
Biomarker Analysis	Non-invasive disease detection	Monitoring disease progression, Treatment response assessment	IBD, Cancer	[51]
Molecular Imaging	High-resolution visualization of tissues	Visualizing inflammation, tissue changes, Disease staging	Gastrointestinal cancers, Inflammation	[52]
Serological Assays	Identification of disease-specific antibodies	Autoimmune gastrointestinal disorder, Infection diagnosis	Celiac disease, Infections	[53]
Capsule Endoscopy	Minimally invasive visualization of the GI tract	Small intestine exploration, Diagnosing obscure bleeding	Small intestinal disorders, Bleeding disorders	[54]
Virtual Colonoscopy	CT scan-based colon imaging without invasive procedure	Colorectal cancer screening, Polyp detection	Colorectal cancer, Polyps	[55]
Breath Tests	Analysis of gases for detecting specific gastrointestinal conditions	*H. pylori* infection, Carbohydrate malabsorption	*H. pylori* infection, Malabsorption	[56]
Stool DNA Testing	DNA analysis from stool samples for colorectal cancer screening	Early detection of colorectal cancer, Adenoma identification	Colorectal cancer, Precancerous lesions	[57]

## 3. Artificial Intelligence in Gastrointestinal Care

Artficial intelligence [AI] technologies introduced invarious medical domains are noticeably transforming the healthcare landscape. Among these, gastroenterology is undergoing a paradigm shift as AI-driven solutions are being used to develop gastrointestinal diagnostics, treatment strategies, and overall patient care [58]. The potential of artificial intelligence in gastrointestinal care stems from its ability to analyse large amounts of patient data, including medical records, imaging studies, and genetic information, to extract meaningful insights. This transformative capability enables early disease detection, accurate diagnosis, individualized treatment, and even disease progression prediction. Furthermore, AI has the potential to alleviate the resource constraints and clinical variability common in the management of GI conditions, thereby standardizing care and improving patient outcomes [59].

Endoscopic AI algorithms can aid in real-time lesion detection and characterization, augmenting the accuracy of procedures such as colonoscopies and esophagogastroduodenoscopies [60]. AI-enabled image analysis in radiology can aid in the detecting subtle abnormalities in radiographic studies, allowing for early diagnosis and intervention [61]. However, incorporating AI into gastrointestinal care presents challenging. AI-powered predictive models have emerged as a promising tool for disease risk assessment, transforming how healthcare professionals identify individuals at high risk of developing various conditions, including GI disorders [62]. These models can analysediverse datasets to create personalised patient risk profiles, encompassing genetic profiles and microbiome compositions, dietary habits and clinical history. Implementing AI-driven predictive models in clinical practice, however, necessitates careful validation, integration into existing healthcare workflows, and transparent patient communication [63].

During endoscopy and radiological imaging, AI algorithms, particularly in image analysis, have demonstrated remarkable accuracy in detecting gastrointestinal lesions, tumors, and polyps. Machine learning models can process large amounts of visual data quickly, assisting healthcare providers in identifying abnormalities that the human eye may miss [64] AI-enabled predictive analytics have the potential to revolutionize gastrointestinal disease management, especially in chronic conditions like IBD/IBS, where therapy response varies greatly between patients [65]. AI-driven treatment optimization reduces the burden on patients and healthcare systems by minimizing trial and error. AI-powered telemedicine platforms and wearable devices enable continuous monitoring of gastrointestinal conditions. Patients can collect data on symptoms, dietary habits, and vital signs, which AI algorithms can analyze for disease activity or medication adherence [66]. The use of AI also accelerates drug discovery by identifying potential therapeutic targets and predicting drug efficacy through molecular and genetic data analysis. This has far-reaching implications for developing novel treatments for GI, including precision medicine approaches [67].

AI and ML-powered diagnostic tools can also help to standardize diagnostic processes. These technologies increase the consistency of diagnoses across different healthcare providers and settings by reducing subjectivity and variability in interpretation [68] In a recent study, analgorithm developed based on specific texture and color filters, combined with ML achieved a sensitivity and specificity of 83% [69]. This algorithm correctly identified early neoplastic lesions from endoscopic images, implying its potential use in clinical settings [70].

## 4. Therapeutic Breakthroughs: Precision Treatment Approaches

**Figure 3:**
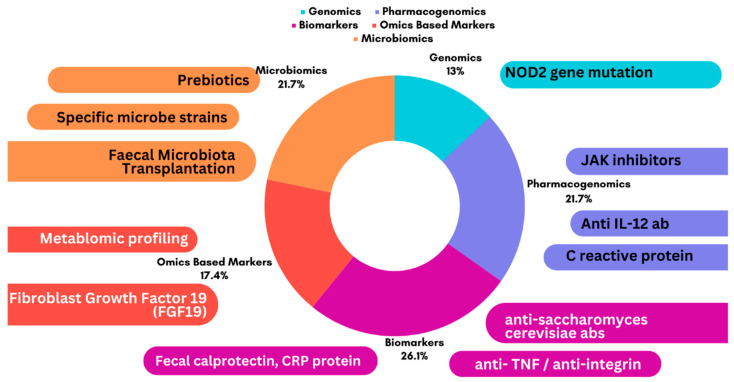
Visual representation of the integration of genetic and microbiome data in personalized treatment strategies.

Precision treatment approaches in gastrointestinal care are ushering a new era of therapy tailored to the unique characteristics of each patient, enhancing treatment outcomes, reducing side effects, and optimising healthcare resource utilization [71]. Several exciting therapeutic breakthroughs have reshaped the scenario of gastrointestinal care, opening new opportunities for both patients and clinicians in recent years [Figure 3]. One promising group of the drugs for GI is Janus kinase [JAK] inhibitors, such as tofacitinib and upadacitinib have shown efficacy in the treatment of moderate to severe ulcerative colitis [72]. Anti-IL-23 and anti-IL-12 antibodies, such as ustekinumab and risankizumab, have shown efficacy in Crohn’s disease [73,74]. Precision medicine aims to provide tailored treatment plans based on an individual’s unique genetic profile. By analyzing an individual’s genetic information, healthcare providers can identify specific gene mutations or variations that may contribute to the development of IBD [75,76].

This information can guide the selection of appropriate medications and dosages, minimizing side effects and maximizing treatment efficacy. One example of genomic testing in IBD is the identification of the NOD2 gene mutation [77]. This mutation is associated with an increased risk of developing Crohn’s disease. By identifying individuals with this mutation, healthcare providers can implement preventive measures and tailor treatment plans to manage the disease effectively. In addition to identifying genetic variations, genomic testing can also predict an individual’s response to specific medications [78].

## 5. Biomarkers in Precision Medicine for IBD

As previously discussed, elevated levels of CRP and fecal calprotectin are associated with active inflammation in the gut, indicating the need for more aggressive treatment approaches. Precision medicine is not limited to clinical practice, it also plays a vital role in advancing research and development [79,80]. Clinical trials focusing on precision medicine in IBD are ongoing, aiming to discover new treatment targets and optimize existing therapies. For example, biologics, such as anti-TNF agents and anti-integrin antibodies, have revolutionized IBD treatment by directly inhibiting the inflammatory cascade. These targeted therapies have shown promising results, inducing and maintaining remission in many patients [81].

The gut microbiome is a collection of microorganisms found in the digestive tract, including bacteria, viruses, fungi, and other microorganisms. These microorganisms are essential for digestion, immune system regulation, metabolism, and overall gut health. Fecal microbiota transplantation [FMT] is a microbiota-based intervention that involves transferring faeces from a healthy donor to a recipient [82]. The procedure aims to restore a balanced, healthy microbiome compositionin the gut. FMT has been studied for recurrent *Clostridium difficile* infections [CDI], IBD, IBS, and metabolic disorders [83]. Rather than using whole faeces, researchers are investigating targeted approaches to modulate the microbiome, including the use of specific bacteria strains and prebiotics [84].

While microbiota-based interventions show promise, several challenges need to be addressed, such as donor screening and long-term effects. It is critical to select appropriate donors for FMT to ensure safety and effectiveness [85]. Donors undergo thorough screening to prevent the spread of infections or other unwanted microbes. The long-term consequences of FMT and microbiome modulation are still being investigated, as altering the gut microbiome may have unintended consequences. FMT is currently regulated by health authorities, and guidelines are evolving as research advances [84].

FMT has shown promising results in managing IBD symptoms and improving overall gut health. Several studies have reported positive outcomes, with individuals experiencing reduced inflammation, improved symptoms, and increased quality of life after FMT [86]. The procedure has also been found to be relatively safe, with a few adverse effects reported. One of the main advantages of FMT is its potential for long-term remission. Unlike traditional treatments for IBD, which may only provide temporary relief, FMT has the potential to induce long-lasting changes in the gut microbiota, leading to sustained improvements in symptoms [87]. This is particularly exciting for individuals with IBD who have not responded well to conventional therapies or who experience frequent flare-ups. However, it is important to note that FMT is still considered an experimental treatment for IBD and is not widely available [88]. Although commercial the shelves product are now available to be used for FMT, There are also risks associated with the procedure, including the transmission of infectious diseases, allergic reactions, and the potential for adverse effects from the donor material. As such, FMT should only be performed by experienced healthcare professionals in specialized settings.

The role of FMT in managing IBD is continuously evolving through ongoing clinical trials. These trials have provided more robust evidence regarding the benefits and risks of FMT in IBD management and have helped identify the most effective methods of administration [89]. Preliminary results from some clinical trials have shown promising outcomes, with a significant proportion of individuals experiencing symptom improvement and disease remission following FMT. However, further research is needed to determine the optimal timing, frequency, and duration of FMT treatment, as well as to identify the specific subgroups of individuals with IBD who are most likely to benefit from this intervention [90].

As the field of microbiota-based interventions and FMT continues to evolve, there is an increasing recognition of their potential role in the management of IBD [91]. These interventions have the potential to complement existing treatment options and provide additional benefits for individuals with IBD. Integrating microbiota-based interventions into IBD treatment plans requires a personalized approach [92]. Healthcare providers need to consider individual factors such as disease severity, subtype, and response to conventional therapies when determining the most appropriate interventions. Additionally, ongoing monitoring and follow-up are essential to assess treatment response and make necessary adjustments to the treatment plan [93,94].

## 6. Advancements in Pharmacotherapy for Gastrointestinal Disorders

Pharmacotherapy for GI disorders has made significant advances, revolutionizing the treatment of a variety of conditions [Figure 3]. These advancements have been driven by a better understanding of the underlying pathophysiology and the pursuit of targeted therapeutic strategies [95]. IBD, which includes Crohn’s disease and ulcerative colitis, has seen the emergence of novel agents. Tofacitinib and upadacitinib, two JAK inhibitors, have shown efficacy in modulating the immune response and alleviating symptoms [96]. Furthermore, the introduction of biological therapies that target specific cytokines, such as interleukin-23 [IL-23] and IL-12, including ustekinumab and risankizumab, has shown promise in inducing remission and maintaining disease control. Serotonin receptor modulators such as alosetron and lubiprostone, provide targeted symptom relief in IBS [97,98].

Extensive research on personalized therapies, focusingon the gut microbiome’s involvement in metabolic and functional diseases, is anticipated [99].

## 7. Gene Therapies and Gene Editing for Inherited Gastrointestinal Disorders

Advances in molecular biology have created unprecedented opportunities for treating inherited GI disorders. Gene therapies and gene editing techniques hold immense promise in addressing the genetic abnormalities underlying various GI disorders [100]. The process of introducing functional genes into patients’ cells to correct or replace malfunctioning genes is known as gene therapy. This approach is particularly promising for inherited GI with well-characterized genetic mutations. For instance, gene therapy aims to deliver functional CFTR [Cystic Fibrosis Transmembrane Conductance Regulator] genes into affected cells in diseases such as cystic fibrosis, where mutations in the CFTR gene cause defective chloride ion transport [101]. Recent advances in viral vector design and delivery systems have increased the efficiency of gene transfer, allowing for more precise targeting of affected tissues [102,103].

CRISPR-Cas9 gene editing techniques have transformed the research or health science field by allowing precise modification of specific DNA sequences. Single-gene mutation-caused GI disorders are prime candidates for gene editing interventions [104]. CRISPR-Cas9 can be employed to correct the genetic mutation responsible for the enzyme deficiency in conditions such as hereditary tyrosinemia type 1, where a deficiency in fumarylacetoacetate hydrolase [FAH] leads to toxic metabolite accumulation [105]. By inserting the correct genetic sequence into patient cells, the underlying metabolic dysfunction can be rectified. Despite their potential, gene therapies and gene editing techniques face several significant obstacles in treating inherited GI [106]. Key challenges include the successful delivery of therapeutic genes or gene editing tools to specific target tissues, minimizing off-target effects, and managing potential immune responses. Additionally, the ethical implications of gene editing and the long-term consequences of genetic interventions require careful consideration. Ongoing research is focused on improving delivery methods, increasing gene editing precision, and ensuring the safety and efficacy of these approaches [107].

Irritable IBS, a gastrointestinal disorder linked to the gut microbiome, that manifests symptoms like constipation, diarrhoea, or both. Recent studies have reported that variations in the normal gut microbiota may contribute to the low-grade intestinal inflammation associated with IBS. Individuals with IBS exhibit an imbalance in the gut microbiomewith elevated levels of harmful bacteria/attachment to the intestine, such as Firmicutes, particularly *Ruminococcin*, *Clostridium*, and *Dorea* while having a significant decrease in specific species such as *Bifidobacterium* and *Fecalibacterium* spp. [108].

The findings on IBS can be leveraged to develop targeted interventions and therapies, such as probiotics or prebiotics, to alter the gut microbiomeandpromote health and prevent IBS symptoms. Colorectal cancer is another gastrointestinal disorder linked to the gut microbiome [CRC] [109]. Research indicates that the gut microbiome may influence the development and progression of CRC. For instance, certain bacterial specieslike *Fusobacteriumnucleatum*, are more abundant in CRC tumours compared to adjacent normal tissue. Additional studies have found that the microbiome can influence the immune response to CRC, thereby influencing tumour growth and progression. Microbiome analysis can help identify specific microbial populations that may be involved in the development and progression of CRC [110].

## 8. Minimally Invasive Interventions: Endoscopic and Surgical Advances

Endoscopic techniques have proven invaluable in the diagnosis of a wide range of GI [33]. Compared to traditional surgical approaches, these minimally invasive procedures offer the advantage of direct visualisation, enabling precise assessment, therapeutic interventions, and reduced patient discomfort. Endoscopic advances have made a significant difference in the diagnosis and treatment of GI [111]. Endoscopy allows for direct visualisation and intervention within the gastrointestinal tract using cutting-edge imaging technologiesand adaptable instruments. Techniques such as endoscopic ultrasound [EUS], capsule endoscopy, and double-balloon endoscopy have improved the accuracy of disease localization and characterization accuracy, facilitating minimally invasive interventions [112,113].

Laparoscopic and robotic-assisted surgeries have significantly transformed gastrointestinal interventions. These approaches offer several advantages over open surgeries, including smaller incisions, less blood loss, and shorter hospital stays [114] With their enhanced dexterity and visualisation capabilities, robotic systems enable surgeons to perform intricate procedures with unparalleled precision. Minimally invasive techniques are now used to treat a wide range of gastrointestinal conditions, including gallbladder removal and hernia repair, as well as complex colorectal resections and bariatric surgeries [115].

Diagnostic endoscopy is essential in the accurate diagnosis of gastrointestinal conditions. Clinicians can directly visualise the mucosal surfaces of the oesophagus, stomach, intestines, and colon using techniques such as esophagogastroduodenoscopy [EGD], colonoscopy, and enteroscopy [115]. These procedures aid in the detecting abnormalities such as ulcers, polyps, and lesions, allowing for early diagnosis and treatment. Advanced imaging technologies, including chromoendoscopy, narrow-band imaging, and confocal laser endomicroscopy, enhance visualisation and tissue characterization, ultimately improving diagnostic accuracy [116].

Moreover, endoscopic interventions have evolved beyond diagnosis to encompass a range of therapeutic procedures. Endoscopic mucosal resection [EMR] and Endoscopic Submucosal Dissection [ESD]enable the removal of precancerous and early-stage cancerous lesions without need for extensive surgery [117]. Endoscopic retrograde cholangiopancreatography [ERCP] can address biliary and pancreatic disorders, facilitating interventions such as stone removal and stent placement. Endoscopic obesity therapies, such as intragastric balloons and endoscopic sleeve gastroplasty, offer less invasive alternatives to surgical weight loss procedures [118]. NOTES [Natural Orifice Transluminal Endoscopic Surgery have the potential to combine the benefits of minimally invasive surgery with a truly scar less approach. This may result in a lower risk of infection, shorter hospital stays, and higher patient satisfaction [119,120].

## 9. Robotic and Laparoscopic Surgery for Improved Outcomes and Reduced Invasiveness

**Figure 4:**
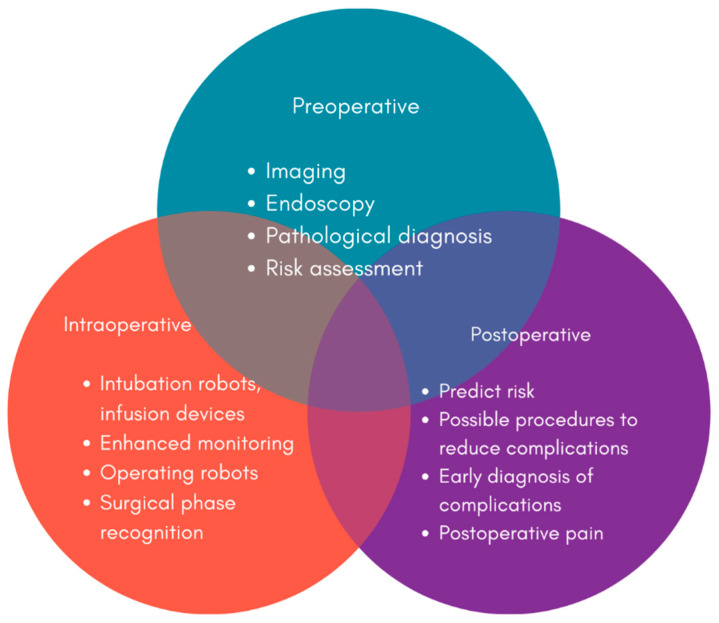
Schematic illustrating the application of artificial intelligence in the preoperative, intraoperative and postoperative stages of the disease.

The fusion of robotic and laparoscopic surgery has heralded a new era of surgical precision and patient-centered care, reshaping the landscape of surgical interventions for a variety of medical conditions, including GI disorders. These minimally invasive approaches provide enhanced visualisation, more precise instrumentation, and reduced tissue trauma than traditional open procedures, resulting in better patient outcomes and shorter recovery times. Robotic-assisted surgery synergizes human surgical expertise with the advanced capabilities of robotic systems [121]. This synergy enables more precise and flexible procedures, particularly beneficial in complex gastrointestinal surgeries like colorectal resections and esophagectomies. Laparoscopic surgery, characterized by small incisions and specialised instruments, further minimizes invasiveness and enhances patient recovery. It has emerged as the gold standard for a variety of gastrointestinal procedures, including cholecystectomy and hernia repair. Laparoscopic techniques reduce postoperative pain, reduce the risk of wound infection, and improve cosmetic outcomes, resulting in higher patient satisfaction [122]. Figure 4 summarizes the application of artificial intelligence in the preoperative, intraoperative and postoperative stages of GI diseases.

## 10. Personalized Medicine in Gastrointestinal Disorders

Personalized medicine has emerged as a paradigm shift in healthcare, with application to GI ailments holding enormous promise for improving diagnosis, treatment, and patient outcomes [71]. As GI disorders encompass a wide range of conditions with varying etiologies, clinical presentations, and treatment responses. Personalized medicine tailors medical care to each patient’s unique characteristics, considering genetics, molecular profiles, lifestyle, and environmental influences [122].

Genetics and genomics have ushered in a new era of personalized treatment strategies, transforming the healthcare setting, particularly in GI medicine. This transformation ensures comprehensive management through the application of artificial intelligence in the preoperative, intraoperative and postoperative stages of the disease [71]. Individual genetic makeup is crucial in determining disease susceptibility, progression, and response to interventions [123]. Understanding these genetic underpinnings has opened up previously unexplored avenues for tailoring treatments to each patient’s specific genetic profile [124]. Genetic and genomic information can reveal a person’s proclivity for GI disorders. Variations in genes linked to IBD, colorectal cancer, and celiac disease provide critical information for determining disease risk. Genetic testing has the potential to identify high-risk individuals, allowing for early interventions and screenings that lead to better outcomes [125].

Comprehensive assessments that delve into a patient’s medical history, genetic predispositions, and biomarker profiles serve as the foundational step for tailoring therapeutics. Patient’s data inform the selection of interventions that offer the highest probability of success for a specific individual. In the case of IBDs, therapies can be personalised based on disease severity, subtype, and genetic markers that influence the treatment response [126]. The integration of omics data, which encompassing genomics, proteomics, metabolomics, and related disciplines, has emerged as a transformative force in precision medicine, especially within the field ofgastroenterology. Omics technologies enable comprehensive profiling of biological molecules and processes, providing unprecedented insight into the intricate molecular mechanisms underlying these disorders [127].

## 11. Future Prospects: Transforming Gastrointestinal Disorder Management

Rapid advances in medical research and technology are driving transformative changes in the management of GI disorders. The future holds promising prospects that could revolutionize diagnostic approaches and treatment strategies [128]. Emerging technologies in diagnostics, such as liquid biopsy and advanced imaging modalities like molecular imaging techniques provide non-invasive means to detect GI disorders early, allowing for proactive interventions before symptoms manifest. Integrating genetic and omics data will almost certainly improve predictive modeling, identifying high-risk individuals and guiding personalized preventive measures [129]. Treatment paradigms are also changing and Precision medicine approaches will guide the development of targeted therapies by considering genetic, molecular and lifestyle factors into account. Immunotherapies, gene therapies, and microbiome-based interventions have the potential to transform the treatment of diseases such a s IBD and diabetes are treated [129].

## 12. Emerging Technologies Such as Nanomedicine and Wearable Devices

Revolutionary technologies are emerging in the healthcare sectors with the potential to reshape GI diagnosis, treatment, and management [130]. Nanomedicine and wearable devices, two notable advancements, are poised to drive significant changes in this domain. Nanomedicine involves the use of nanoparticles at the nanoscale to target specific cells, tissues, or disease processes [131]. This approach promises highly targeted drug delivery in GI, reducing side effects and increasing therapeutic efficacy. Nanoparticles can be engineered to deliver medications directly to affected sites, overcoming the challenges of traditional drug distribution and improving treatment outcomes. Wearable devices, which enable continuous monitoring of vital signs, physiological parameters, and disease-specific markers, are another significant development [132,133]. In the context of GI health, these devices can track dietary habits, monitor symptoms, and provide early warnings of disease flare-ups, allowing for timely interventions and personalized treatment adjustments [134].

## 13. Utilization of Telemedicine and Remote Patient Monitoring

Integrating telemedicine and remote patient monitoring has emerged as an effective treatment strategy for GI, and redefined the interaction and collaboration between patients and the healthcare system. These technologies improve accessibility, convenience, and personalised care, particularly for people facing challenges due to geographic distance, mobility, or chronic medical conditions [135]. Telemedicine facilitates remote consultations between patients and healthcare professionals, reducing barriers to healthcare access. Patients with GI disorders can consult specialists, discuss symptoms, and receive treatment recommendations in the most comfortable setting possible [136]. Telemedicine platforms enable real-time communication, ensuring timely responses to concerns and changes to treatment plans [137].

## 14. Potential of 3D Printing in Customizing Treatment Solutions

3D printing has caused a paradigm shift in healthcare by enabling unprecedented patient-specific treatment solutions, particularly in GI [138]. This technology has the potential to completely transform the design and manufacture of medical devices, implants, and anatomical models, allowing for more tailored interventions and better patient outcomes [139,140]. The transformation of gastrointestinal disorder management through novel therapies necessitates a critical examination of the ethical and social implications of these advancements [141,142]. Informed consent emerges as a critical component, requiring a comprehensive understanding of the intricacies, risks, and potential outcomes associated with novel treatments by the patient. Equitable access to these therapies is imperative, considering the possible disparities in affordability and availability that may exacerbate healthcare inequalities [143]. Furthermore, the unknown long-term effects of interventions such as gene editing and microbiota-based therapies raise ethical concerns about patient well-being. The careful selection of donors for fecal microbiota transplantation (FMT) warrants ethical scrutiny, encompassing considerations of donor influence on recipients and the ramifications of such interactions [144].

3D-printed anatomical models enable surgeons to practice complex procedures prior to surgery [145,146,147]. Additionally, 3D printing facilitates the creation of custom-made medical devices and implants that precisely match an individual’s anatomy in GI applications [148]. Patients requiring gastrointestinal stents or prosthetics, for example, can benefit from devices that are specifically designed to fit their unique anatomical structures, reducing the risk of complications and enhancing treatment efficacy [148].

## 15. Future Prospects and Challenges

Advances in genetic and epigenetic research have opened up new avenues for investigation the causes and potential treatments for functional GI. Researchers are exploring the hypothesis that environmental factors during foetal development significantly contribute tolater susceptibility to chronic diseases. This fetal developmental plasticity is thought to be mediated by epigenetic changes, such as DNA methylation and histone modification. Functional GI [FGIDs], such as IBS and functional dyspepsia [FD], are caused by a complex interplay of factors and pathways [as shown in Table 2]. Consequently, these conditions are challenging to treat, presenting significant obstacles to the pharmaceutical industry in developing effective treatments [95,145]. These factors can range from a simple failure to recognize the severity of the condition to more complex issues that hinder and pose challenges indiagnosis, pathophysiology, outcome measures, and drug development [146]. As advanced technologies and novel interventions continue to shape the landscape of gastroenterology, ethical considerations become more complex and essential to providing responsible patient care [147]. The intersection of cutting-edge advancements and ethical principles presents both exciting opportunities and significant challenges that must be navigated carefully.

## 16. Conclusions

Advancements in medical research, technology, and innovative treatment modalities have yielded remarkable progress in the management of GI disorders in recent years. This review examines the transformative developments in diagnosis, treatment methodologies, and management of GI disorders, emphasizing their significant impact on patient care and the promising future prospects they offer. Additionally, the ascendancy of personalized medicine, propelled by genomic and molecular research, holds the potential to revolutionize treatment strategies by customizing them to the unique genetic profiles of individual patients. From the introduction of endoscopic procedures to the advent of minimally invasive surgeries, the landscape of GI disorder management has undergone significant evolution. Imaging modalities such as MRI and CT scans have accelerated diagnosis, rendering it faster and accurate. Furthermore, the rise of personalized medicine, driven by genomic and molecular research, holds the potential to transform treatment strategies by customizing them to the unique genetic profiles of individual patients.

These advancements have profoundly benefited patients suffering from IBD, IBS, and GERD. Innovative pharmaceuticals, biologics, and targeted therapies have not only provided relief but also significantly enhanced the quality of life for countless patients. Meanwhile, telemedicine and digital health platforms have improved patient-provider interactions by enabling remote monitoring and expanding access to patient care. Looking ahead, the prospects for GI disorder management appear promising. Artificial intelligence and machine learning algorithms are poised to improve diagnostics, predict disease progression, and optimize treatment plans. CRISPR gene editing holds the potential to correct genetic abnormalities associated with certain GI disorders, thereby opening the door to long-term cures.

Despite the considerable progress made, issues such as equitable access to cutting-edge treatments, the ethical considerations surrounding genetic manipulation, and the necessity for ongoing clinical trials to validate novel approaches persistently raise concerns. Collaboration among healthcare providers, researchers, and policymakers will be essential in addressing these challenges and ensuring benefits for all patients. Indeed, the future holds significant promise for more precise, personalized, and effective treatments.

## Figures and Tables

**Table 2:** Roadmap depicting the potential trajectory of advancements in gastroenterology over the next decade.

Disease Type	Molecular Mechanism	Pathway Involved	Treatment Options	Target Drugs	Significance	Reference
IBD [IBD]	Dysregulated immune response to gut microbiota	TNF-alpha signaling, IL-23/Th17	Biologics [anti-TNF, anti-IL-23]	Infliximab, Vedolizumab	Revolutionized IBD management, improved quality of life	[16,81]
Gastroesophageal Reflux Disease [GERD]	Weak lower esophageal sphincter, acid reflux	Esophageal motility dysfunction	Proton pump inhibitors [PPIs]	Omeprazole, Esomeprazole	Alleviates symptoms, prevents complications	[148]
Celiac Disease	Immune reaction to gluten in the small intestine	Immune-mediated pathways	Gluten-free diet	None [dietary management]	Avoids long-term complications, improves health	[146]
Colorectal Cancer	Genetic mutations, abnormal cell growth	Wnt signaling pathway	Surgery, chemotherapy, radiation	Oxaliplatin, Fluorouracil	Early detection and treatment improve survival	[147]
IBDs [IBS]	Altered gut-brain communication, motility issues	Serotonin signaling, gut-brain axis	Dietary modifications, medications	Antispasmodics, Linaclotide	Enhances quality of life, symptom management	[31]
Peptic Ulcer Disease	*H. pylori* infection, acid erosion of stomach lining	*H. pylori* infection, acid production	Antibiotics, proton pump inhibitors	Amoxicillin, Omeprazole	Prevents complications, promotes ulcer healing	[148]

## Data Availability

Not applicable.
